# A novel method for the evaluation of proximal tubule epithelial cellular necrosis in the intact rat kidney using ethidium homodimer

**DOI:** 10.1186/1472-6793-7-1

**Published:** 2007-02-23

**Authors:** Joshua R Edwards, Evangelos A Diamantakos, Jacob D Peuler, Peter C Lamar, Walter C Prozialeck

**Affiliations:** 1 Department of Pharmacology, Midwestern University, 555 31st Street, Downers Grove, IL 60515, USA

## Abstract

**Background:**

Ethidium homodimer is a cell-membrane impermeant nuclear fluorochrome that has been widely used to identify necrotic cells in culture. Here, we describe a novel technique for evaluating necrosis of epithelial cells in the proximal tubule that involves perfusing ethidium homodimer through the intact rat kidney. As a positive control for inducing necrosis, rats were treated with 3.5, 1.75, 0.87 and 0.43 mg/kg mercuric chloride (Hg^2+^, intraperitoneal), treatments which have previously been shown to rapidly cause dose-dependent necrosis of the proximal tubule. Twenty-four h after the administration of Hg^2+^, ethidium homodimer (5 μM) was perfused through the intact left kidney while the animal was anesthetized. The kidney was then removed, placed in embedding medium, frozen and cryosectioned at a thickness of 5 μm. Sections were permeabilized with -20°C methanol and then stained with 4',6-diamidino-2-phenylindole (DAPI) to label total nuclei. Total cell number was determined from the DAPI staining in random microscopic fields and the number of necrotic cells in the same field was determined by ethidium homodimer labeling.

**Results:**

The Hg^2+^-treated animals showed a dose-dependent increase in the number of ethidium labeled cells in the proximal tubule, but not in other segments of the nephron. Other results showed that a nephrotoxic dose of gentamicin also caused a significant increase in the number of ethidium labeled cells in the proximal tubule.

**Conclusion:**

These results indicate that this simple and sensitive perfusion technique can be used to evaluate cellular necrosis in the proximal tubule with the three-dimensional cyto-architecture intact.

## Background

As a highly perfused organ having unique filtration, secretory and reabsorptive functions, the kidney is susceptible to toxic injury. Of the various segments of the nephron, the proximal tubule is especially prone to toxic injury. As a result of its location adjacent to the glomerulus and the presence of specific secretory systems for organic acids and bases, the proximal tubule is frequently exposed to higher levels of toxicants than other segments of the nephron and is, therefore, often a primary site of nephrotoxic injury. In light of its importance as a site of toxic injury, considerable attention has been focused on the development of sensitive and accurate methods for quantifying cell injury and cell death in the proximal tubule. Obviously, a key issue in attempting to quantify toxic injury in the proximal tubule is to identify dead or dying cells. The techniques that have been used to accomplish this fall into three categories: those based on changes in general cell morphology; those based on biochemical markers in the cascade of events leading to oncotic/necrotic or apoptotic cell death; and those based on a loss of cell membrane integrity [1-4].

Morphological analysis of whole kidney tissue is a simple and widely used method to determine the nephrotoxic effects of xenobiotics while keeping the three dimensional cyto-architecture of the kidney intact [5,6]. However this technique is semi-quantitative at best, with a necrotic/apoptotic score or index assigned to a certain degree of cell death or tissue damage. Ultimately this method is largely based on an observer's judgment as to whether a cell is dead or alive.

Several methods of identifying cell death in the kidney are based on specific markers of key biochemical events in the process of necrosis/oncosis or apoptosis, for reviews see [2,3,7]. For example, DNA fragmentation is one defining characteristic of apoptotic cell death and assays based on the Terminal Deoxynucleotidyltransferase-Mediated UTP End Labeling (TUNEL) and electrophoretic analysis of DNA have been primarily used to detect apoptotic cell death [8-10], although the TUNEL assay does not always discriminate apoptotic from necrotic or autolytic cell death [11]. Other assays have utilized specific markers of key events in the process of apoptosis or necrotic cell death. Some of the markers on which these assays are based include the mitochondrial membrane permeabilization, the translocation of phosphatidyl serine from the inner to the outer cell surface, and the activation of caspase or calpain enzymes [12-14]. These assays have the advantage of indicating the exact stage of progression of cell death. However, these assays can not always be used in the intact kidney and they can be difficult to interpret. Furthermore, the assays may be subject to interferences by the toxic substances themselves [15,16].

Some of the most widely used methods for assessing viability are based on the loss of cell membrane integrity that occurs prior to cell death [17]. Many of these methods based on cell membrane permeability involve the detection of cytosolic enzymes such as lactate dehydrogenase (LDH) and alkaline phosphatase that appear in the urine as the apical membrane of the epithelial cells of the proximal tubule begin to break down. These enzymatic assays are rapid, quantitative, and relatively easy to perform. However, many of the enzymatic urinary markers of cell death are not cell- or even organ-specific. For example, with toxic agents such as cadmium, that cause hepatic as well as renal damage, urinary enzymatic markers of cell death are not an ideal method to determine renal damage. Furthermore, certain substances can inhibit or interfere with the actual enzyme activity. For example, the nephrotoxic metal Hg^2+ ^directly inhibits LDH activity [18]. Recently, several commercially–available ELISA-based assays have been developed for the determination of urinary alpha-glutathione-S-transferase, a urinary marker that is thought to be specific for proximal tubule damage [19]. However these products are rather expensive and have yet to be fully validated for use in assessing the effects of most nephrotoxic substances.

Other techniques to assess cell necrosis through the loss of cell membrane integrity involve the use of cell membrane-impermeable dyes such as trypan blue, or fluorochromes such as various ethidium or propidium compounds [15,20]. These agents are normally excluded from live cells but can label dead or dying cells in which cell membrane integrity is compromised. While these methods have been widely used for assessing necrotic cell death in *in vitro *model systems they have been less widely used for *in vivo *studies.

While considering the assessment of renal viability in an intact kidney it is important to acknowledge work done by Molitoris and colleagues and Peti-Peterdi and co-workers, who have pioneered the use of multiple photon fluorescence microscopy to study renal physiological and biochemical processes *in vivo *[20-22]. This powerful technique involves the use of nuclear fluorochromes and allows for the real time quantification of apoptotic and necrotic renal cell death in the intact kidney in a live animal [23]. However this technique requires considerable technical expertise along with the use of very expensive equipment. Moreover, the optical constraints limit its use to the visualization of functional changes only in the outermost areas of the exposed regions of the kidney.

As part of our ongoing research to identify the mechanism underlying the nephrotoxic effects of cadmium (Cd^2+^) and other metals, we have developed a novel *in situ *renal cell viability assay that involves perfusing the fluorescent cell viability marker, ethidium homodimer through the intact rat kidney. Ethidium homodimer is a nuclear fluorochrome with high affinity for DNA (Ka = 2 × 10^8 ^M^-1^), a molecular weight of 856.8 and low membrane permeability [24]. Traditionally, ethidium homodimer has been widely used as an indicator of necrosis of cells in culture. However it has also been used as a marker of necrosis in specific populations of cells within isolated corneae [25] and in whole lung preparations [26-28].

To validate the sensitivity and utility of this assay, rats were treated with known nephrotoxic doses of Hg^2+^. Hg^2+^primarily accumulates within and targets the epithelial cells of the proximal tubule, especially at the junction of the cortex and medulla [29-31]. Exposure to nephrotoxic concentrations of Hg^2+ ^results in renal dysfunction as indicated by elevations in blood urea nitrogen (BUN) and serum creatinine [32]. At the cellular level, Hg^2+ ^exposure is associated with oxidative stress, decreased reduced glutathione levels [33], alterations in stress protein expression [29,34] and alterations in cell adhesion molecule expression and localization [32]. Acute exposure to nephrotoxic doses of Hg^2+ ^results primarily in necrosis with apoptotic cell death occurring to a lesser extent at very high doses of Hg^2+ ^[35]. In order to further evaluate the utility of this technique for assessing pathological changes in the proximal tubule, additional animals were treated with the aminoglycoside antibiotic gentamicin at a dose (100 mg/kg for 8 consecutive days) that has previously been shown to cause necrosis of the proximal tubule [36]. In addition, other animals were treated with the nephrotoxic metal Cd^2+^, using a subchronic dosing protocol (0.6 mg/kg 5 days per week for 6 weeks) that causes dysfunction of the proximal tubule without causing extensive tubular necrosis [37-39].

The results of these studies show that perfusion of the whole kidney with ethidium homodimer allows for the accurate determination of cell death in conditions where the three-dimensional cyto-architecture of the kidney remains intact.

## Results

No animals died in any of the treatment groups. However, animals in the 3.5 mg/kg Hg^2+ ^treatment group appeared lethargic with orange-colored bristly fur, especially around the neck at 24 h following Hg^2+ ^exposure. Others have noted similar responses in rats using similar doses of Hg^2+ ^[29]. In general, the abdominal cavity of animals dosed with 3.5 mg/kg HgCl_2 _tended to have visceral connective tissue that appeared to be dehydrated or "sticky". Subsequently the clearing of connective tissue from the aorta and kidneys for the *in situ *viability assay was more difficult and required slightly more time in the higher Hg^2+^-treatment groups, as compared to control animals. Macroscopic observations of the unperfused, right kidney demonstrated a pale coloration of the cortex and medulla in animals treated with 3.5 mg/kg Hg^2+^.

### Effects of Hg^2+ ^on serum and urinary parameters

**Figure 1:**
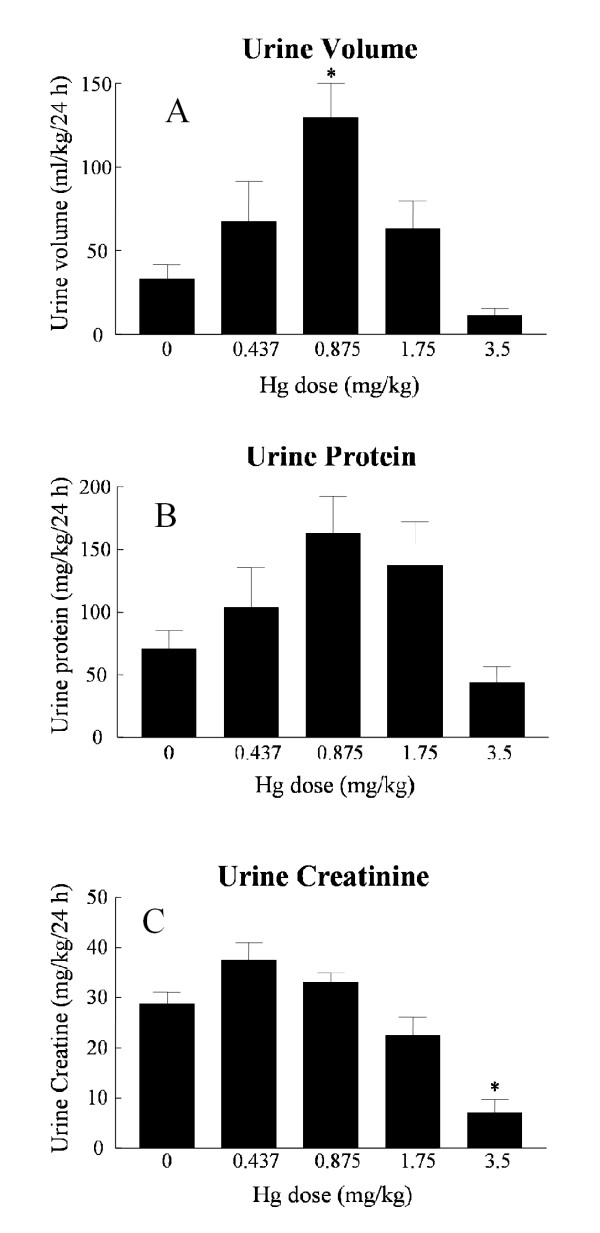
Effects of Hg^2+ ^on urine volume (A), urinary protein (B) and urinary creatinine (C). Animals received single, ip injections of varying Hg^2+ ^doses (0.437, 0.875, 1.75 and 3.5 mg/kg) and 24 h urine volume samples were collected and analyzed for creatinine and protein as described under methods. The values for each data point represent the mean ± SE for a total n ≥ 6 for each treatment group. An asterisk (*) indicates significant difference from control (one-way ANOVA, P < 0.05, post-hoc Tukey's test).

**Figure 2:**
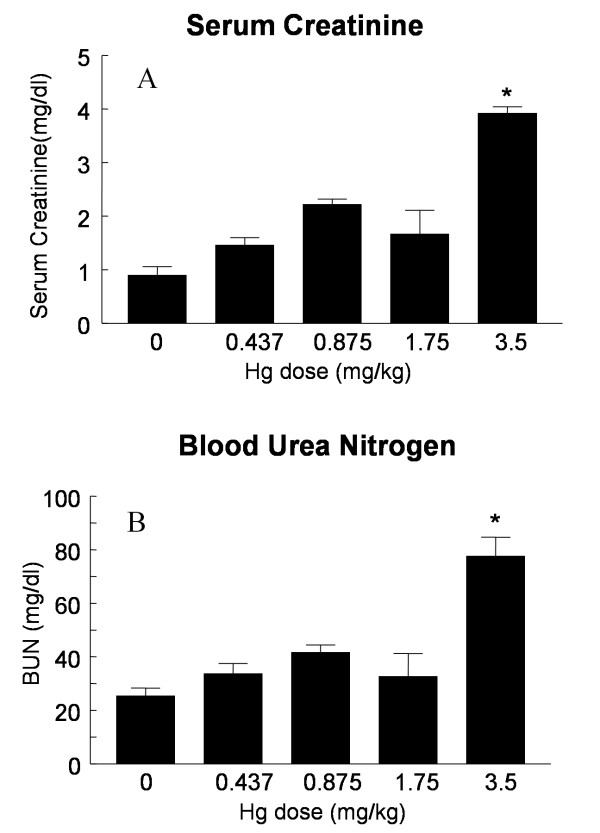
Effects of Hg^2+ ^on serum creatinine (A) and BUN (B). Animals received single, ip injections of varying Hg^2+ ^doses (0.9% NaCl vehicle control, 0.437, 0.875, 1.75 and 3.5 mg/kg) and 24 h blood samples were collected and analyzed for BUN and serum creatinine. The values for each data point represent the mean ± SE for a total n ≥ 6 for each treatment group. An asterisk (*) indicates significant difference from control (one-way ANOVA, P < 0.05, post-hoc Tukey's test).

Hg^2+ ^treatment had a biphasic, dose-dependent effect on urine volume (Fig. 1A). Moderate doses of Hg^2+ ^(0.875 mg/kg) caused an increase in urine volume. However, the highest doses of Hg^2+ ^(3.5 mg/kg) resulted in a trend of decreasing mean urine volume values that was not statistically significant. The urinary excretion of protein also showed a trend toward this type of dose-dependent, biphasic response, although differences in mean values did not reach levels of statistical significance (Fig. 1B). Treatment with the highest dose of Hg^2+ ^caused a significant decrease in the urinary excretion of creatinine (Fig. 1C). This high dose of Hg^2+ ^also caused a significant increase in serum creatinine and BUN (Figures 2A and 2B). The results in Figures 1(A–C) and 2(A, B) are similar to results that have been reported by other investigators using similar Hg^2+^-treatment protocols in rats [32,40] and indicate that after 24 h the highest doses of Hg^2+ ^caused severe acute renal failure and the lower doses caused less severe impairment of renal function.

**Figure 3:**
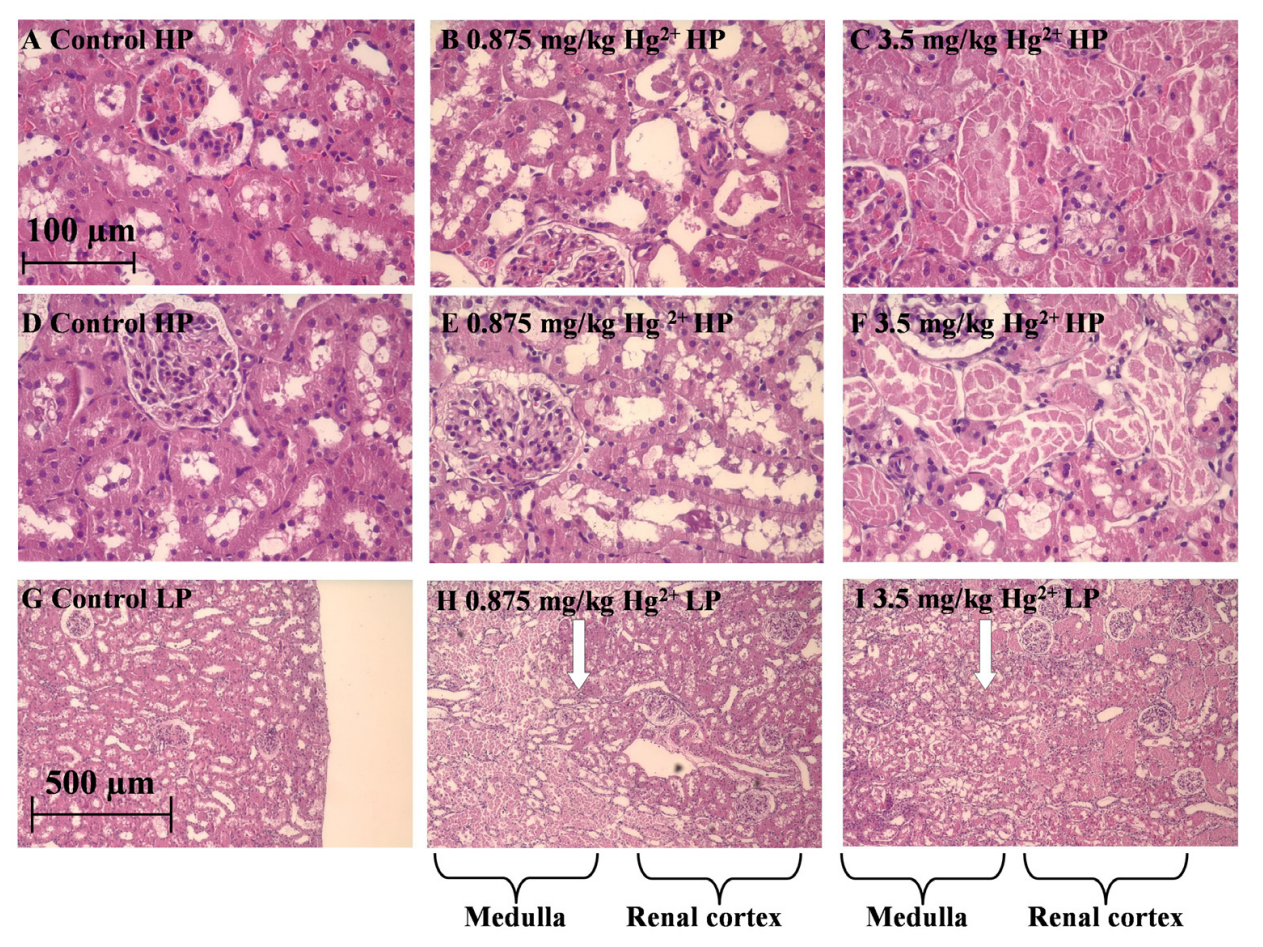
The effects of Hg^2+ ^on the general morphology of the outer renal cortex. Rats were treated with Hg^2+ ^(0.9% NaCl vehicle control, 0.875 and 3.5 mg/kg, i.p.). 24 h later, the kidneys were removed, and processed for H & E staining. Panels A-C are representative sections from the non-perfused right kidney and panels D-F are representative sections from the perfused left kidney under high-power (HP). (G-I) are representative images of the perfused left kidney under low-power (LP). Panels A, D, G are from vehicle control treated animals, panels B, E, H are from 0.875 mg/kg Hg^2+^-treated animals and panels C, F, I are from 3.5 mg/kg Hg^2+^-treated animals. A white arrow in panels H and I represent the boundary between the renal cortex and the outer medulla. The scale bar in the top left image represents 100 μm and the scale bar in the bottom left image represents 500 μm.

Figure 3 shows H & E stained sections of representative samples of the outer renal cortex from control and Hg^2+^-treated animals. Figures 3(A–C) shows images of the non-perfused right kidney taken with a 40× objective. The epithelial cells of the proximal tubules of control animals (3A) exhibited regular cuboidal shapes, well-defined nuclei and were closely associated with adjacent cells. Panels D-F of Figure 3 show images of comparable fields from the perfused left kidneys from the same animals from which the panels A-C (non-perfused right kidney), were obtained. As expected, the perfused, left kidneys all contained fewer blood cells than the corresponding non-perfused right kidneys. However, in all other respects, the perfused kidney showed the same morphologic properties as the corresponding non-perfused kidneys; the samples from the Hg^2+^-treated animals showed evidence of dose-dependent necrosis in the proximal tubule. Furthermore, perfused (3D) and non-perfused (3A) kidneys of control animals showed no signs of cell death or damage. This indicates that the perfusion technique did not cause any morphologic changes in the proximal tubules. Images taken with a 10× objective (Fig. 3H) show that at a dose of 0.875 mg/kg Hg^2+ ^the boundary between the cortex and outer medulla is more distinct compared to the 3.5 mg/kg Hg^2+ ^dose (Fig. 3I). In the images taken with a 40× objective, samples from the 0.875 mg/kg Hg^2+^-treated animals (Fig. 3B, 3E) some swelling of epithelial cells in the proximal tubule was apparent coupled with a modest loss of nuclei. However, epithelial cells appeared to remain attached to the basement membrane. In samples from the 3.5 mg/kg Hg^2+^-treated animals (Fig. 3C, 3F) the epithelial cells throughout the proximal tubule were swollen and appeared to slough off from the basement membrane. Furthermore, the 3.5 mg/kg Hg^2+^-treated animals demonstrated a loss of nuclei with widespread degeneration and necrosis. In general, the glomeruli and distal tubules from the Hg^2+^-treated animals showed normal morphology. These effects of Hg^2+ ^are similar to those reported in other studies [32,34,41] and indicate that Hg^2+ ^caused extensive necrosis of the proximal tubule but had little effects on other segments of the nephron.

### Effects of Hg^2+ ^on ethidium-labeling in the renal cortex

**Figure 4:**
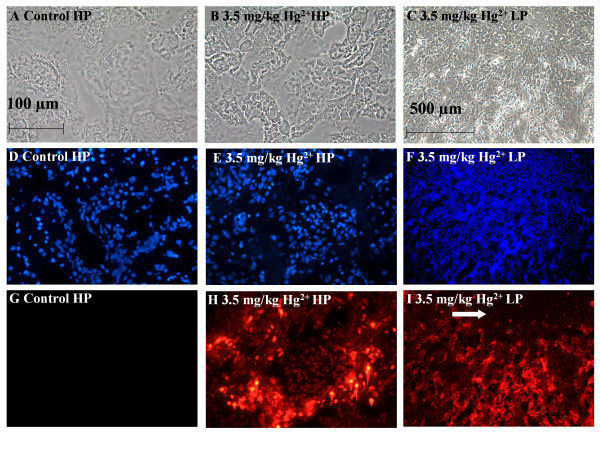
Effects of Hg^2+ ^on ethidium-labeling in the renal cortex. Animals were treated with Hg^2+ ^(3.5 mg/kg, ip) for 24 h and the left kidneys were infused with ethidium homodimer. Cryosections of the kidneys were then fixed, permeabilized and labeled with DAPI as described in the Methods section. Panels A, D, G are high-powered (HP) images from control animals and panels B, E, H are from 3.5 mg/kg Hg^2+^-treated animals. Panels C, F, I represent low-powered (LP) images from 3.5 mg/kg Hg^2+^-treated animals. Panels A-C show phase-contrast images of the same fields in D-F and G-I, respectively. Panels D-F show total nuclei labeled in each field by DAPI, G-I show labeled nuclei by ethidium homodimer. The white arrow in panel I indicates the boundary between inner medulla and the renal cortex. The scale bar in the top left image represents 100 μm and the scale bar in the top right image represents 500 μm.

Cryosections of the ethidium-perfused kidneys from control and Hg^2+^-treated animals were fixed, permeabilized and stained with DAPI, as described in the Methods section. Figure 4 shows representative images that were captured from random fields of the renal cortex of control and Hg^2+^-treated animals. Figure 4A–C show the sample fields under phase contrast imaging. Figure 4D–F show the DAPI labeling and Figure 4G–I show the ethidium labeling. As expected, there was almost a complete absence of ethidium labeling in the nuclei of the samples from control animals (Fig. 4G). However, animals treated with 3.5 mg/kg Hg^2+ ^show a significant increase in the number of ethidium-labeled cells in the proximal tubule (Fig. 4H). Low powered (10× objective) images of 3.5 mg/kg Hg^2+^-treated animals (Fig. 4I) show that the ethidium-labeling was confined to the renal cortex, and outer medulla, but was absent in the inner medulla (arrow) these results are in agreement with previous studies showing that the proximal tubules are the primary site of Hg^2+ ^toxicity [30,31,35].

### Dose-response relationship for the effects of Hg^2+ ^on proximal tubule epithelial cell viability

**Figure 5:**
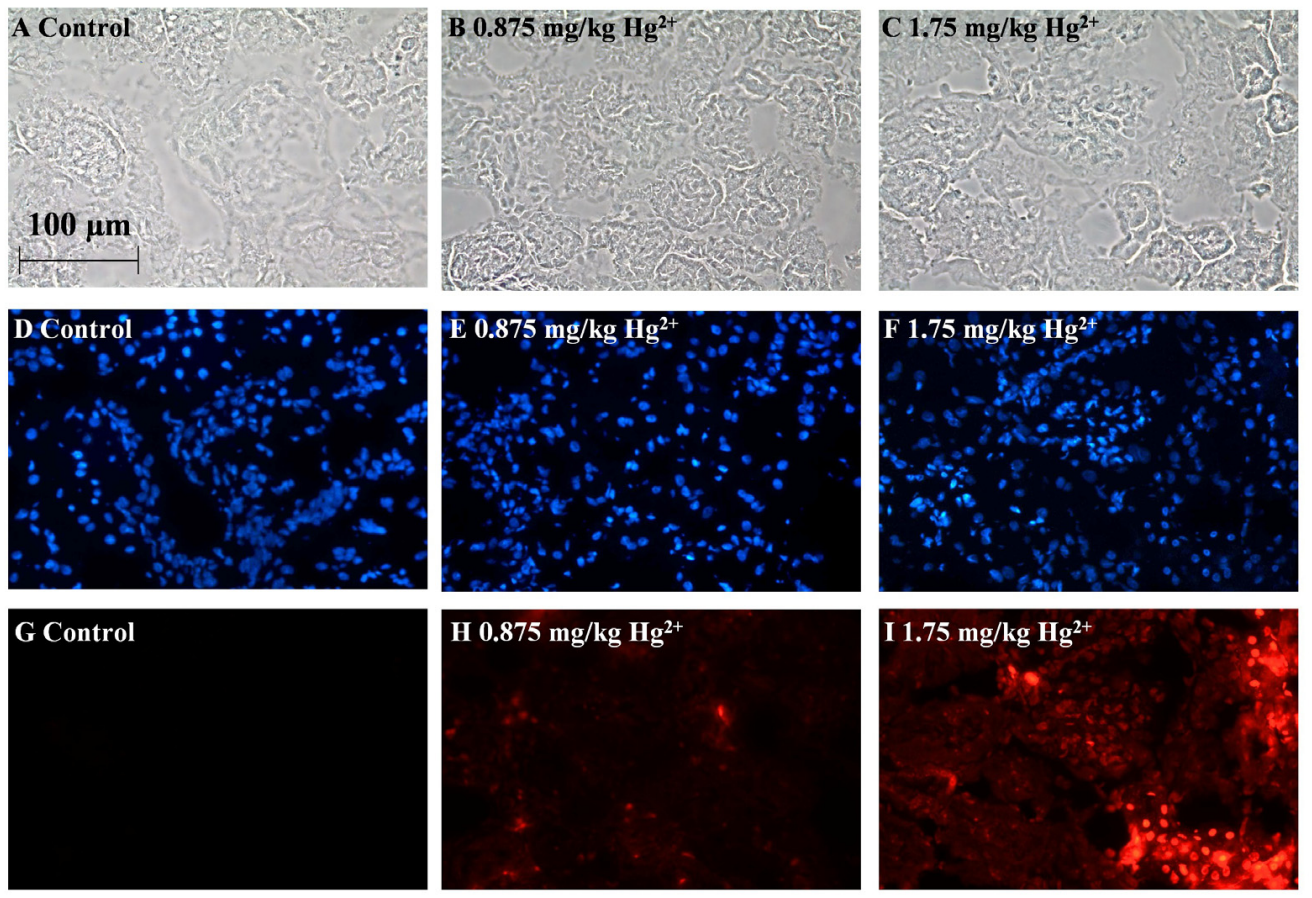
Dose-response for Hg^2+^-induced cell death. Animals were treated with Hg^2+ ^(0.875 and 1.75 mg/kg, ip) for 24 h and the left kidneys were infused with ethidium homodimer. Cryosections of the kidneys were then fixed, permeabilized and labeled with DAPI as described in the Methods section. Panels A, D, G are images from 0.9% NaCl vehicle control-treated animals. Panels B, E, H are from 0.875 mg/kg Hg^2+^-treated animals, and panels C, F, I are from 1.75 mg/kg Hg^2+^-treated animals. Panels A-C show phase-contrast images of the same fields in D-F and G-I, respectively. D-F show total nuclei labeled in each field by DAPI fluorescence, G-I show nuclei labeled by ethidium homodimer. The scale bar in the top left image represents 100 μm.

**Figure 6:**
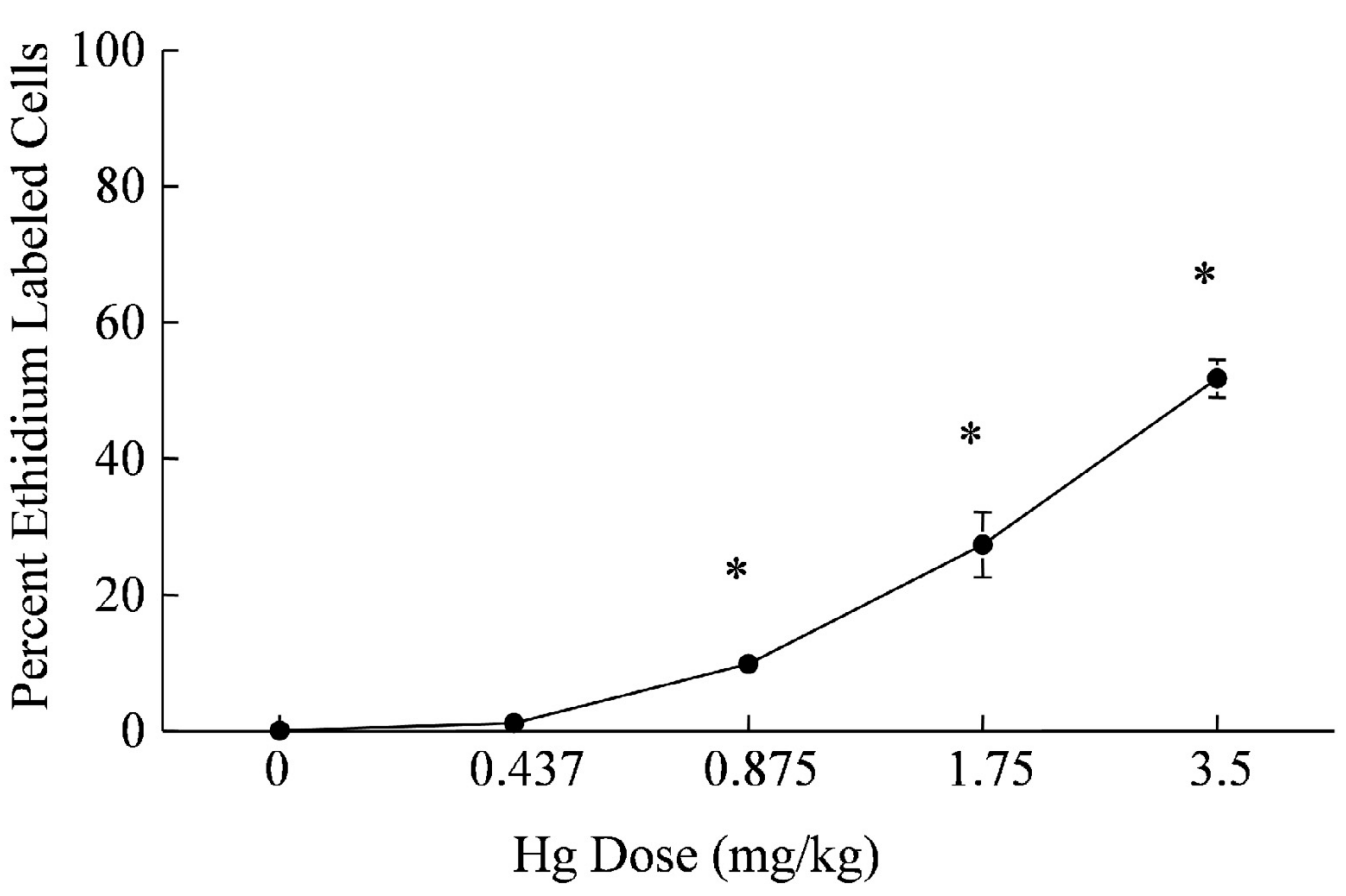
Dose-dependent increase in the percent ethidium-labeled proximal tubule epithelial cells in the renal cortex following treatment with Hg^2+^. Digital images from three fields per microscope slide with a total of four slides from each animal were quantified for necrosis analysis. Each animal represents one n value or replicate, in each treatment group. The values for each data point represent the mean ± SE for a total of n ≥ 6 for each treatment group. Asterisk (*) indicates significant differences from 0.9% NaCl vehicle control treatment group (one-way ANOVA, p < 0.001, post-hoc Tukey's Test).

To test the sensitivity of the *in situ *viability assay, animals were treated with varying doses of Hg^2+ ^(0.4375, 0.875, 1.75 and 3.5 mg/kg). The results are summarized in Figure 5. Figure 5A–C shows the sample fields under phase contrast imaging. Figure 5D–F shows the DAPI labeling and Figure 5G–I shows the ethidium labeling. There was almost a complete absence of ethidium labeling in the nuclei of the samples from control animals (Fig. 5G). However, at Hg^2+ ^doses of 0.875 (Fig. 5H) and 1.75 mg/kg (Fig. 5I) there was an increase in the number of ethidium-labeled nuclei of proximal tubule epithelial cells in the renal cortex compared to images from control animals (Fig. 5G). The dose-response relationship of Hg^2+^-induced renal cell death is shown graphically in Figure 6. Note that the lowest dose of 0.4375 mg/kg Hg^2+ ^did not cause a significant increase in the number of ethidium-labeled cells but the higher doses, 0.875, 1.75 and 3.5 mg/kg showed significant dose-dependent increases in the percent of ethidium-labeled cells. This dose-response of Hg^2+^-induced renal necrosis is consistent with the morphological observations described in Fig. 3 and is similar to those reported in other studies using non-quantitative or semi-quantitative morphological analyses [31,33,35,42].

### Effects of gentamicin and Cd^2+ ^on renal cell viability

**Figure 7:**
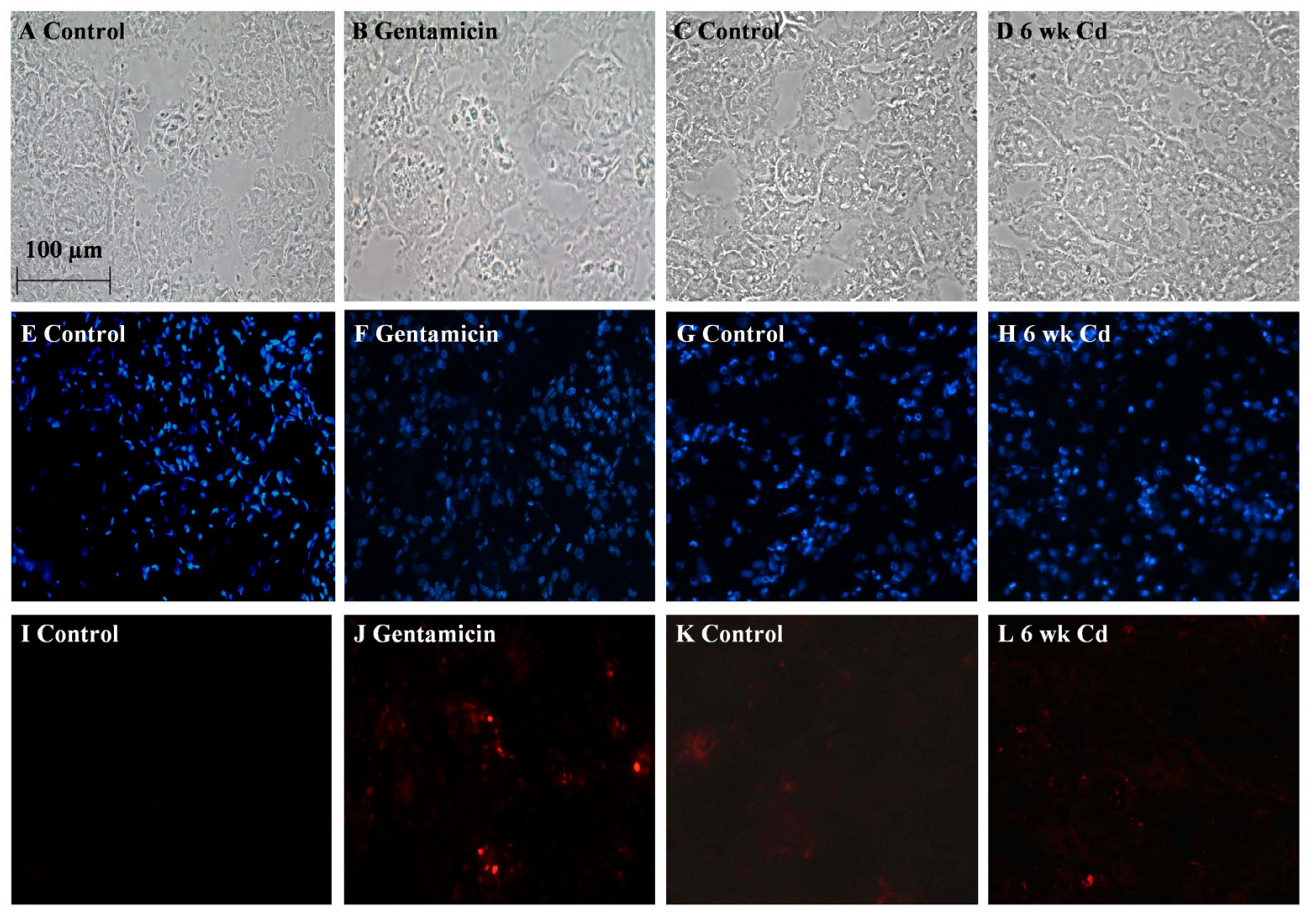
The effects of gentamicin and Cd^2+ ^on cell membrane integrity. Animals were treated with either Cd^2+ ^(0.6 mg/kg, sc 5 days a week for 6 weeks) or gentamicin (100 mg/kg ip per day for 8 days) and the left kidneys were infused with ethidium homodimer. Cryosections of the kidneys were then fixed, permeabilized and labeled with DAPI as described in the Methods section. Panels A-D are phase contrast images corresponding to DAPI-labeled panels E-H and ethidium homodimer labeled nuclei in panels I-L. Gentamicin treatment resulted in increased ethidium homodimer labeling (J) in the renal cortex as compared to control (I). No differences in ethidium homodimer labeling were detected in 6-week Cd^2+^-treated images (L) compared to control (K). The scale bar in the top left image represents 100 μm.

In order to further evaluate the utility of this technique for assessing pathological changes in the proximal tubule, additional animals were treated with the aminoglycoside antibiotic gentamicin (n = 2) at a dose that has previously been shown to cause necrosis of the proximal tubule [36]. Animals treated with gentamicin for 24 h excreted larger volumes of urine (mean values of 34.1 and 140.9 ml/kg/24 h for control and gentamicin-treated, respectively) and displayed elevations in urinary protein content (mean values of 34.8 and 172.9 mg/kg/24 h for control and gentamicin-treated, respectively). These findings are similar to those reported in the literature [43] and are characteristic of gentamicin nephrotoxicity. In addition, other animals were treated with the nephrotoxic metal Cd^2+^, using a sub-chronic dosing protocol (0.6 mg/kg, sc, 5 days per week for 6 weeks) that has previously been shown to cause dysfunction of the proximal tubule without causing tubular necrosis [37-39]. Urine samples from 6 week Cd^2+^-treated animals showed significant proteinuria (38.6 ± 4.1 and 50.5 ± 3.8 mg/kg/24 h for control and Cd^2+^-treated (n = 12), respectively) and decreased body weight gain. However, there were no changes in urine volume or creatinine excretion (data not shown). Figure 7 shows the results of the renal cell necrosis assay in samples from animals that had been treated with either gentamicin (Fig. 7J) or Cd^2+ ^(Fig. 7L). Animals treated with the proximal tubule-specific nephrotoxic, gentamicin, demonstrated a significant increase in the percent of ethidium homodimer labeled cells (Fig. 7J). In the samples from the gentamicin-treated animals 21 ± 9% of cells were labeled with ethidium as opposed to < 1.0% in the control samples (n = 3 fields from 2 control and 2 gentamicin-treated animals). None of the samples from the Cd^2+^-treated animals exhibited signs of necrosis in the renal cortex (Fig. 7L). This is in agreement with previous studies using the identical treatment protocol [38].

## Discussion

In examining the nephrotoxic effects of xenobiotics, one of the most important endpoints that investigators must evaluate is cell death. Unfortunately, the tasks of identifying and quantifying dead or dying cells in a complex organ such as the kidney are not as easy as they may appear to be at first glance. In the current report, we describe a relatively simple procedure for identifying and quantifying necrotic cells in the proximal tubule that involves the perfusion of the intact kidney with ethidium homodimer and the subsequent observation of fluorescence of cryosections from the renal cortex. Furthermore, we show that this technique can be used to detect changes in cell viability resulting from other site-specific nephrotoxicants such as gentamicin. This would indicate the possible utility of employing this *in situ *viability assay for a wide variety of potential nephrotoxicants.

This method offers a couple of significant advantages over many of the other techniques that have been used to assess the cell viability/cell death in the intact kidney. First, while the method does require some surgical preparation of the animal, it is relatively easy to perform and it does not require the use of elaborate or expensive equipment. Secondly, the labeling of cell nuclei with ethidium homodimer provides a very clear-cut end point for identifying necrotic cells. A random analysis of the pixel density indicated that the intensity of light emission of almost all of the ethidium homodimer labeled cells was at least 10–100 times the intensity of background labeling (data not shown). There were few, if any, cells that exhibited intermediate levels of labeling. This makes it easy for the observer to interpret the results; the nuclei are either labeled or they aren't and the delineation is very clear cut. Also, since the delineation between live and necrotic cells is so clear cut, it should be possible to interface this technique for use with quantitative image analysis and statistical programs.

The renal perfusion technique that was used in these studies is similar to renal perfusion procedures that have been used by others to harvest renal epithelial cells for cell culture or to infuse markers for the evaluation of renal function [44-46]. In the procedure described in this study, the ureter is sectioned and the perfusion pressure (50–100 mmHg) is below that of reported rat systolic blood pressure [47]. This low pressure tubular perfusion procedure has little effect on general renal morphology (see Fig. 3). Since the perfusion procedure washes most of the blood cells from the kidney, the morphology and cyto-architecture of the proximal tubules in the perfused kidneys is actually clearer and better preserved than in non-perfused kidneys. Since the morphology of the kidney is so well-preserved, it should be possible to adapt this procedure to include fixatives and labels or markers of specific molecules and physiologic functions of interest.

One caveat to keep in mind regarding this method is that it is based on the loss of cell membrane integrity, which is a relatively late event in the process of cell death [48]. It does not assess the early stages of cellular injury. This is a significant issue because an increasing volume of evidence indicates that some nephrotoxic substances can have profound effects on renal function, without causing necrosis. One example of such a substance is Cd^2+^. The results of our studies utilizing this new technique confirm results of previous studies from this and other laboratories [37-39] indicating that subchronic exposure to Cd^2+ ^for 6 weeks causes proximal tubule dysfunction without causing significant necrosis of tubular epithelial cells. It should be noted that some studies have shown that this Cd^2+ ^treatment protocol causes a limited level of apoptosis in the proximal tubule [37,39]. While we did not specifically address the issue of Cd^2+^-induced apoptosis in the present study, we feel that this method could eventually be adapted for this purpose. In this procedure, ethidium homodimer, which is infused through the intact kidney, labels necrotic cells preferentially. However the DAPI staining occurs after the cells are permeabilized with methanol and labels all necrotic, apoptotic and unaffected cell nuclei. Apoptotic nuclei appear to have a distinctive fragmented or diffuse appearance that distinguishes them from necrotic or normal cells [5,49,50]. Therefore through close examination of nuclei labeled with DAPI [51], it may be possible to determine the ratio of necrotic to apoptotic cells using this method. It should also be noted that while the present studies focused on the use of ethidium homodimer to evaluate cell death in the proximal tubule, the technique has the potential to identify cellular necrosis in other segments of the nephron.

Another notable limitation of this technique is that it essentially provides a "snapshot" image of cellular necrosis in the kidney at a single point in time. Obtaining data for multiple time points requires the use of multiple animals. This is in contrast to the more sophisticated multiple photon imaging techniques that allow for real time imaging of the kidney of a single animal over time [20-22]. Despite this shortcoming, the ethidium homodimer labeling technique is relatively easy to perform, inexpensive and should provide a useful means for quantifying necrosis in the proximal tubule and, possibly, other segments of the nephron.

## Conclusion

In this study, we describe a novel renal assay to detect necrosis that involves the infusion of ethidium homodimer into the intact rat kidney. Results of this study indicate that this simple and sensitive perfusion technique can be used to evaluate necrosis in the proximal tubule with the three-dimensional cyto-architecture intact.

## Methods

### Materials

HgCl_2 _(#M1136), creatinine (#C4255), sterile 0.9% sodium chloride (#8776), CdCl_2 _(#C3141), gentamicin (#G3632) and other chemicals were of high purity and were purchased from Sigma Chemical Company (St. Louis, MO). Ethidium homodimer-1 (#E1169) and 4',6-diamidino-2-phenylindole (DAPI) (#D1306) was purchased from Invitrogen, Eugene, OR. Coomassie^® ^Plus Protein Assay Kit (#23236) and bovine serum albumin (#23209) were purchased from Pierce, (Rockford, IL). Saturated picric acid solution (#80456) was purchased from Fluka Biochemika (Buchs, Switzerland).

### Animals, Hg^2+^, Cd^2+ ^and gentamicin treatment protocols

Adult male Sprague-Dawley rats were purchased from Harlan, (Indianapolis, IN) and were randomly assigned to the Hg^2+^-treated or control (sterile 0.9% NaCl) groups (n ≥ 6 for all treatment groups). Rats were given a single intraperitoneal (ip) injection of Hg^2+ ^at doses of (0.4375, 0.875, 1.75, 3.5 mg/kg) in sterile 0.9% NaCl solution. The varying Hg^2+ ^doses correlate to 1.6 μmol, 3.2 μmol, 6.5 μmol and 12.9 μmol Hg^2+^/kg body wt, respectively. Control animals received a single ip injection of the 0.9% NaCl vehicle alone. The total volume of fluid injected for Hg^2+ ^and control animals was less than 1 ml. After animals were dosed with either Hg^2+ ^or vehicle NaCl, they were placed in individual metabolic cages, and 24 h urine samples were collected. To further validate the utility of this *in situ *viability assay, we examined changes in proximal tubule cell viability in male Sprague-Dawley rats after 6-week sub-chronic Cd^2+ ^(n ≥ 6) exposure and acute gentamicin dosing (n = 2). Briefly, animals were treated by subcutaneous injection (sc) of CdCl_2 _at a Cd^2+ ^dose of 0.6 mg/kg in sterile 0.9% NaCl, 5 days per week, for 6 weeks. This dose correlates to 5 μmol Cd^2+^/kg body wt and has been shown to cause proximal tubule dysfunction without causing significant necrosis of the proximal tubule epithelium [38]. Animals in the Cd^2+^-control group received daily, equal volume, sc injections of 0.9% NaCl alone. Gentamicin-treated animals were given daily injections of gentamicin at 100 mg/kg (71.9 μmol/kg) for eight consecutive days. Gentamicin control animals were given ip injection for 8 days with vehicle alone of physiological saline solution, (PSS). This dosing protocol has been shown to cause proximal tubule damage in rats [36].

### Urinalysis

Following each of the treatment protocols the animals were placed in individual metabolic cages and 24 h urine samples were collected. The urine volumes were recorded and the urine samples were aliquoted and stored at -80°C until analyzed for protein and creatinine content. Urinary creatinine was determined by a modified form of the colorimetric method of Shoucri and Pouliot [52]. Briefly, 3 ml of a working reagent that consisted of 0.21 M NaOH and 2.4 mM picric acid was added to 20 μl urine samples. After 10 min, the absorbance at a wavelength of 505 nm was measured. Urine creatinine values were determined based on the resulting absorbances of known creatinine standards and were expressed as mg/kg/24 h. Urinary protein was determined by the method of Bradford [53] using the Coomassie^® ^Plus Protein Assay kit (Pierce #23236).

### Determination of blood urea nitrogen (BUN) and plasma creatinine

Serum creatinine was assayed using the same method as described above for urinary creatinine. BUN was assayed following the protocol of Talke and Schubert [54].

### In situ evaluation of cell viability

The animals were anesthetized [ketamine/xylaxine (67/7) mg/kg]. The abdominal cavity was opened and the left kidney was isolated from surrounding connective tissue. The aorta was freed from the surrounding connective tissue and from the adjacent vena cava. A 4.0 silk ligature (Roboz Surgical, Gaithersburg, MD) was positioned around the aorta as far caudally as possible (approximately 2 cm below left renal artery) and tied immediately. A second ligature was position around the aorta just above the left renal artery and tied just prior to the insertion of the catheter. In order to allow for an isolated perfusion of the left kidney, a third ligature was tied around the right renal artery and vein to prevent perfusion of the right kidney, just prior to insertion of the perfusion catheter. The perfusion catheter was fashioned from a 23 gauge stainless steel needle and connected to polyethylene (PE 50) tubing filled with a solution of 5 μM ethidium homodimer in PSS which contained (mM): 115.0 NaCl, 5.5 glucose, 16.0 NaHCO_3_, 1.0 MgCl_2_, 0.2 Na_2_HPO_4_, 0.8 NaH_2_PO_4_, 1.0 CaCl_2_, and 5.0 KCl. An incision was made approximately half way through the aorta just above the lower ligature and the catheter was inserted into the aorta and advanced to a position just below the left renal artery. The catheter was tied and secured in place with a new ligature. At this time a blood sample was taken from the inferior vena cava. Once the catheter was secured, the ureter was sectioned and ethidium homodimer was perfused through the kidney at a flow rate of 1 ml/min for 5 min then increased to 3 ml/min for 5 min. The pressure of the perfusate was 50 mmHg at a flow rate of 1 ml/min and 100 mmHg at 3 ml/min, as measured in preliminary experiments with a low pressure transducer (Grass P10EZ) attached to a Model 7 Grass polygraph (Grass Technologies/Astro-Med, Inc., West Warwick, R.I.). The left kidney was then perfused with PSS at 3 ml/min for 10 min to wash out any residual or unbound ethidium homodimer. The perfused left kidney was removed, decapsulized, cut through the transverse plane into three sections and immediately frozen separately at -80°C for later cryosectioning. The non-perfused right kidney was also removed and processed for histological and biochemical analyses. Animals were then euthanized by exsanguination and pneumothorax while under anesthesia.

The frozen left kidney samples were cryosectioned at a thickness of 5 μm. The sections were then mounted on glass slides, fixed and permeabilized in -20°C methanol, and stained with 0.3 μM DAPI, to label all nuclei. The labeled sections were then covered with Aqua Polymount (Polysciences, Warrington, PA) and viewed with a Nikon Eclipse 400 fluorescence microscope within 24 h of DAPI staining. Fields were viewed under both phase contrast and fluorescent illumination using a 40×, high power objective or 10× low power objective. The number of total nuclei and ethidium-labeled nuclei within the field of view of a 40× objective, were determined from random fields within the renal cortex. Each field contained approximately 300 cells in an area of 9.1 × 10^4^μm^2^. Digital images from three fields per slide with a total of four slides from each animal were quantified for viability analysis. Digital images were captured with a Spot digital camera (Diagnostic Instruments, Sterling Heights, MI) using automated exposure times and gain settings for the bright-field images. The dark-field fluorescence images of ethidium homodimer (excitation = 528 nm; emission = 617 nm) and DAPI (excitation = 358 nm; emission = 461 nm) stained slides were captured at a gain setting of 4 and 1 s exposure for green, red and blue. The digital images were processed using Image-Pro Plus imaging analysis software package (Media Cybernetics, Silver Spring, MD). In performing these studies, we found that once the tissue is frozen in embedding medium and stored at -80°C, the ethidium labeling is very stable with no loss of fluorescence over at least 2 months. However, once the tissue sections are cut with a cryotome, washed, stained with DAPI then stored at room temperature there was a subsequent loss of fluorescence over 1–4 weeks. Accordingly, all samples were examined within 48 hours following sections and DAPI labeling.

### Statistical Anaylsis

Data were analyzed using the SigmaStat^® ^statistical program (SPSS Inc., Chicago, IL). Statistical differences were determined using one-way or two-way analysis of variance (ANOVA) where appropriate. If significant differences between sample means were detected (p < 0.05), a post-hoc Tukey-Kramer test was performed to ascertain which mean values were significantly different from control values.

## List of abbreviations

Affinity constant (Ka), analysis of variance (ANOVA), blood urea nitrogen (BUN), Cadmium (Cd^2+^), 4',6-diamidino-2-phenylindole (DAPI), intraperitoneal (ip), lactate dehydrogenase (LDH), Mercury (Hg^2+^), physiological saline solution (PSS), subcutaneous (sc), Terminal Deoxynucleotidyltransferase-Mediated UTP End Labeling (TUNEL)

## Authors' contributions

WCP was responsible for the overall design of the study and the analysis of the results. JDP provided technical guidance and advice in the development of the methods for the renal perfusion of the viability marker. PCL performed most of the biochemical and routine histological analyses. EAD performed most of the renal perfusions and the compilation of the cell viability data. JRE assisted with all phases of the study and wrote most of the manuscript. All authors have read and approved the final manuscript.
